# Knowledge, attitudes, and uptake of mental health services by secondary school students in Gweru, Zimbabwe

**DOI:** 10.3389/fpsyg.2023.1002948

**Published:** 2023-02-01

**Authors:** Sibusiso Khombo, Kennedy Khombo, Robert Shalom Stoddart, Innocent Sifelani, Theresi Sibanda

**Affiliations:** ^1^Department of Applied Psychology, Midlands State University, Gweru, Zimbabwe; ^2^Department of Psychology, University of Limpopo, Limpopo, South Africa; ^3^Centre for Film and Media Studies, University of Cape Town, Cape Town, South Africa; ^4^Department of Psychology, Manicaland State University of Applied Sciences, Mutare, Zimbabwe

**Keywords:** mental health, adolescents, attitudes, help seeking behavior, psychological distress, knowledge, students

## Abstract

**Introduction:**

The study sought to investigate and examine knowledge, attitudes, and uptake of mental health services by secondary school students in the Gweru district.

**Methods:**

Using a qualitative approach, 15 students from three secondary schools in Gweru were purposively sampled for inclusion in this study. Data collection was through semi-structured interviews. The study utilized thematic data analysis and the following themes emerged from the study; ignorance, misinformation, indifference, lack of trust, gender differences, and alternative support.

**Results:**

Generally, the research findings revealed that most secondary school students were aware of the existence of mental health services although they had distorted information on the same. Poor utilization of services was largely attributed to the consideration of “formal” mental health services as the last resort for remedy after the failure of “informal” services to yield positive results. Thus, mental health services were not on the priority list of intervention measures used by secondary students in light of mental health issues. The study recommends psycho-education sessions to promote the utilization of mental health services by secondary school students.

**Discussion:**

Notably, the current study revealed that participants lacked correct information about MHS and its related utility. Munson et al. (2009) concur by stating that some adolescents believed that their mental health challenges such as mood disorders were chronic, and as such, they thought that any form of intervention could not control or remedy their illness.

## Introduction

There is a growing mental health concern among secondary school students because of an upsurge in affective and behavioral disorders. Despite these mental health challenges and the availability of school-based interventions, there is limited or no uptake of these services by secondary school students to improve their mental health. The heightened increase of mental health challenges among students has been attributed to a variety of factors from academic pressure to experienced psychobiological developmental milestones. In England, NHS Digital (2018) reports that one in eight of 5–19-year-olds suffered from an identifiable mental health disorder. In general, available literature has put across that mental health disorders usually commence in adolescents, and the mean age for the onset of mental health disorders is occurring at the age of 14 years (Harris, 2018).

An overarching argument is that secondary school students fall in the adolescent demographic group, which is an important developmental period and should have age-appropriate interventions to deal with their mental health issues (Barrow and Thomas, 2022). Based on the observation that students spend a greater part of the day time at school than in any other formal organizational structure (Rutter et al., 1979, as cited in Fazel et al., 2014), it must be noted that the school may resultantly be a source of stress and support to secondary students. As a source of stress, secondary schools are usually a turning point and exert a lot of pressure on students such as in determining career paths, social relationships, and family expectations and their maturity is tested among many other sources of anxiety (Vaez and Laflamme, 2002; Güneri, 2006). The experienced stress has detrimental effects on affected students. For example, mental distress is one of the reasons for poor academic performance (Vaez and Laflamme, 2008).

The top 10 reasons for help-seeking behavior in schools are communication problems, adjustment to a balance between adulthood and childhood life, romantic relationships, depression, anxiety disorders, test anxiety, study skills, academic failure, low self-esteem, and relation with parents (Güneri, 2006). Given the challenges experienced by students, it is proposed that secondary schools should play a central role in the early detection and provision of services to deal with mental health challenges faced by adolescents (Thorley, 2016; Barrow and Thomas, 2022).

Failure to balance developmental changes, social and psychological needs, and academic pressures may lead students to seek other coping mechanisms which may include drug and substance abuse, alcohol misuse, and contemplation of suicide (Duane et al., 2003). In this same vein, statistics obtained by Pelzer in 2009 as part of the Global School-Based Health Survey from six countries, namely, Zimbabwe, Zambia, Uganda, Kenya, Namibia, and Swaziland indicated that 6.6% of youths misuse alcohol and 10.5% abuse illicit substances (Pelzer, 2009).

There is a growing concern that the limited uptake by secondary students of mental health interventions such as counseling services has an impact on their overall wellbeing, academic achievement, and educational outcomes. It has been observed that counseled students are socially and emotionally balanced (DeStefano et al., 2001), does not drop out of school (Sharkin, 2004), and are more likely to complete school successfully (Rickinson, 1998). Among several other strategies to deal with students’ mental health issues, school-based approaches have an important role as they aim to minimize stigma, reduce practical barriers, promote connectedness, and help-seeking behaviors (Barrow and Thomas, 2022).

All Zimbabwean primary and secondary schools have Guidance and Counseling establishments (Director’s Circular Number 23, 2005), which, among several other functions, cater to students’ mental health needs. While schools could have a positive impact on students’ mental health by becoming centers for mental healthcare (Froese-Germain and Riel, 2012), there are several students who are not accessing early intervention services. Hence, the study’s aim is to investigate the knowledge, attitudes, uptake of mental health services (MHSs) by secondary students, and subsequently probe measures to improve and increase the use of MHS.

Rickwood et al. (2005) divided help-seeking behaviors into two categories, namely, informal (family and friends) and formal (therapists or counselors and community agencies) clusters, the latter of which is the subject of the present research.

## Review of literature

Burke et al. (2008) contend that students displayed negative views toward mental health services, particularly with respect to psychiatric hospitals and medication. The display of negative views is partly attributed to the idea that students did not have information about whom to contact if they had depression and were very aware of the stigma of going to see a “professional” (Burke et al., 2008). Nepal et al. (2020) noted that the majority of the students studying at the senior secondary level were found to exhibit negative attitudes toward mental illness. Although no variables were found to be contributing toward such perceptions, Sartorius (2007) assert that stigma is one factor that tends to promote negative attitude toward people suffering from mental illness due to the lack of knowledge and misinformation, which subsequently leads to prejudice and discriminatory tendencies.

In contrast, a study conducted by Tesfamariam et al. (2018) revealed that a typical secondary school student has an ambivalent attitude toward mental illness. Possibly, this could be a result of the accumulative impact of stigmatizing negative attitudes learned from elders or strengthened by sociodemographic features. Another study conducted among Nigerian secondary school students revealed that just over half (55%) of the students had a positive attitude toward mental illness (Omi Jack Ide et al., 2016). Some of the proposed reasons for negative attitudes include the style of upbringing (Javed et al., 2006) and inadequate information about the illness (Dessoki and Hifnawy, 2009). For example, Burke et al. (2008) are of the view that numerous students did not recognize depression as being a mental health issue and could not distinguish between depression and feeling sad.

It can be concluded that the lack of knowledge and negative attitudes toward mental illness could have an adverse effect on the uptake of MHS based on the theory of planned behavior (Ajzen, 1991), which suggests that attitude is one factor that influences the intention to behave in a certain manner.

Several factors come into play with regard to mental health help-seeking behaviors by secondary students. Rickwood and Thomas (2012) define help-seeking for mental health problems as “an adaptive coping process that is the attempt to obtain external assistance to deal with mental health concerns” (p.180). This sought-after assistance entails both formal (e.g., health services) and informal (e.g., friends and family) sources of help. Unrau and Grinnell (2005) weighs in by contending that help-seeking behaviors involve a request for assistance from informal supports or formalized services for the purpose of dealing with emotional, behavioral, or health problems.

Despite the need for MHS to cater to the needs of affected students, it appears that the students are not motivated to access the services. Burke et al. (2008) observe that although the rate of suicide among young male participants is on the increase, it has been noted that young men are reluctant to utilize mental health services. Furthermore, a study by MacKenzie et al. (2006) suggests that negative attitudes associated with psychological openness might promote men’s underutilization of mental health services. In contrast, it has been revealed from a meta-analysis of coping studies that female participants use more emotion-focused strategies, such as seeking emotional support from others than male participants (Tamres et al., 2002). This means that females may be more willing to seek assistance from others as compared to males.

A study conducted by Burke et al. (2008) indicates ignorance and lack of appreciation of mental illness, combined with an adverse opinion against mental health professionals and fear of stigma as key barriers to access to mental health services, especially, young men. In concurrence, a Canadian survey conducted by Froese-Germain and Riel (2012) revealed that stigma related to accessing MHS is a barrier to the uptake of services by students.

In their New Zealand study conducted in 2007, Lucassen et al. (2011) found that students who were mainly attracted to same-sex individuals experienced difficulties in getting support for emotional worry. As compared with their heterosexual peers, one of the reasons which make it difficult for non-heterosexual students to access MHS is the experienced discouragement emanating from the fact that the students tend to be treated harshly, wherein they are subjected to verbal and physical abuse by both adults and peers (Safren, 1999, as cited in Lucassen et al., 2011).

Studies show that female participants exhibit positive attitudes toward seeking professional psychological help as compared with their male counterparts (Gonzalez et al., 2005; MacKenzie et al., 2006). Concomitantly, Munson et al. (2009) indicated that while not statistically significant, there is an observation that female participants reported higher scores on psychological openness and help-seeking tendency than male participants. In contrast, male participants may feel that society expects them to be strong and get better on their own (Munson et al., 2009), hence their poor help-seeking behaviors.

The Children’s Commissioner in England notes that not all adolescents have access to children and adolescent mental health services (CAMHSs), and receive the mental health services and the support they need (Children Commissioners, 2016). To this end, 79% of CAMHS stated that they imposed constraints and thresholds on adolescents accessing their services, and this meant that unless their cases were severe they were not able to access services. Velasco et al. (2020) concur by suggesting that, to a lesser extent, there are problems that speak to the availability of services and personnel and other structural factors (such as cost, transportation, and waiting times), which are barriers to help-seeking. This means that there are “non-personal” challenges that discourage the uptake of MHS by adolescent students.

Conclusively, it is noted that although adolescents are in dire need of mental health services, Velasco et al. (2020) observe that they are unwilling to seek assistance. In their systematic review study, Velasco et al. (2020) found the following as likely barriers to the uptake of mental health services by adolescents, stigma, family beliefs, and mental health literacy. In addition, Munson et al. (2009) opined that the more adolescents believed their mental health challenges, such as mood disorders, were long-lasting, the less they believed that any form of remedy could control their illness. Therefore, it can be ascertained that several factors come into play in light of help-seeking behaviors by secondary students.

Education stakeholders such as school teachers, parents as well as mental health professionals should aim at equipping children with appropriate knowledge on mental illness (Nepal et al., 2020), as this would improve their attitude toward mental illness (Jyothi et al., 2015), and hence the subsequent uptake of related services. Thus, the implementation of attitude enhancing programs by launching anti-stigmatizing programs and providing appropriate information to secondary-level school students has been shown to yield positive results (Del Casale et al., 2013).

Another possible strategy that has shown effectiveness in an endeavor to promote the uptake of MHS is to implement education programs in schools to curb stigma. As knowledge is related to desirable attitudes, such programs may increase knowledge about mental illness and treatment and, as a result, improve the attitudes toward mental health services among adolescents in general and male participants in particular, (Chandra and Minkovitz, 2007).

In an effort to promote the uptake of MHS by students, Velasco et al. (2020), in their systematic review study, proposed strategies such as psycho-education, peer training interventions, and multimedia interventions. Most of the studies made use of psycho-education and classroom-based interventions. Although all the interventions had a bias toward encouraging help-seeking behaviors, the emphasis and content varied among them, including general mental health topics. Peer training interventions are focused on the training of peers who act as ambassadors of change and social interactions incorporated into the daily activities within the school environment.

Finally, Velasco et al. (2020) propose six types of multimedia interventions that have been developed to address some of the barriers to accessing the adolescent population, such as fear of confidentiality breaches, stigma, and self-reliance. These interventions included interactive films to engage students with mental health-related topics and online platforms providing personalized information regarding the decision-aid process.

## Methods

In an effort to investigate the knowledge, attitudes, and uptake of MHS by secondary students, the study utilized the qualitative research approach. A simple guide to qualitative (n.d.) notes that the qualitative approach seeks to explain people’s thoughts, experiences, beliefs, and attitudes. Given that the study explored the knowledge and attitudes of participants, it meant that the qualitative approach was the most suitable approach for this study.

The use of the purposeful sampling method was guided by the need to select information rich-participants (Patton, 2002) who are knowledgeable about the study area (Cresswell and Plano Clark, 2011). Furthermore, the choice of the sampling method allowed for the selection of available and willing participants who are also able to clearly articulate their opinions and experiences (Bernard, 2002) on MHS.

### Participants and study setting

Fifteen (15) secondary students were selected, and the sample size was determined by the data saturation point, which was reached when no new insights were recorded (Hennink and Kaiser, 2022), and this also signified the collection of adequate information to make necessary conclusions. The study participants were drawn from purposively sampled three secondary schools in Gweru urban district, Zimbabwe. This study utilized 15 participants drawn from target secondary schools in the Gweru district. Of the sampled lot, 46.7% (*n* = 7) were male participants, while 53.7% (*n* = 8) were female participants. The choice of the three study sites was influenced by their ease of accessibility to the lead researcher.

### Data collection tool

A semi-structured interview protocol was developed and utilized for this study. The design of the interview guide was influenced by the study’s focus areas, that is, knowledge, attitudes, and uptake of MHS. The utilized data collection tool promoted the gathering of open-ended data and the exploration of participants’ beliefs, thoughts, and feelings with regard to the phenomena under study (DeJonckheere and Vaughn, 2019).

### Data collection procedure

The researchers made prior arrangements with participants with regard to the date, time, and place for the interviews. On agreed dates and times, one on one interviews were conducted within the premises of identified secondary schools, and the interviews were conducted over 3 days. Each interview ranged between 20 and 40 min, and all responses were tape-recorded. Participants were allowed to use the language they were comfortable with. Transcription of the recordings was done in English to allow for data analysis.

### Ethical considerations

Clearance to undertake this study was obtained from the Midlands State University. Permission to use the school premises and access students was granted by the authorities in concerned schools. Information pertaining to the study was presented to study participants. More importantly, participants were made aware of their right to withdraw from the study at any point without facing any censure or penalties. Furthermore, the confidentiality and privacy of participants’ information during the entire process of the study were guaranteed. To ensure anonymity, the participants were allocated pseudonyms. Following the researchers’ explanation pertaining to the research study and the addressing of participants’ questions, written assent was sought from the participants.

### Data analysis

The six-phased thematic analysis method was used to analyze the gathered data (Braun and Clarke, 2021). First, data familiarization was done, wherein repeated reading of the dataset to promote in-depth familiarity with the data was conducted. Second, codes were generated in which data items related to research questions were coded. In the third phase, coded data were reviewed, analyzed, and merged according to shared meanings culminating in the generation of themes. Phase four of the data analysis looked at the repetitive review of themes *viz*-à-vis coded data items, with themes that did not address research questions being discarded. The fifth phase is about defining and naming themes. It is at this stage that each theme was expressed in relation to both the dataset and research questions. The final phase is about report production and this stage, among other things, addressed the order in which the themes were presented. The use of thematic analysis in this study allowed the determination of participants’ views, opinions, knowledge, and experiences (Caulfield, 2022) on mental health services.

## Findings

This study’s themes were identified under the three key categories, which are (a) knowledge of mental health services (MHSs), (b) attitudes toward MHS, (c) uptake of MHS, and (d) strategies for improving uptake of mental health services.

### Category 1: Knowledge of mental health services

#### Ignorance/lack of awareness

Participants professed ignorance of the usefulness of mental health services (MHSs). The students held varied insinuations, which pointed to their being unaware of the importance of MHS. This is illustrated by the quotations later:

*If I need some to talk to I talk to my boyfriend who is always there to offer me advise and comfort when I am stressed so I don’t think I need a counselor or a psychologist to deal with my problems. (Participant 2)*

*… I do not care about telling someone my problems, I am old enough to deal with my problems and I have never heard of anyone who went to see a counsellor because they are sad or unhappy. For, talking to a stranger is a non-starter. I have enough friends and family to talk to should there be need… (Participant 6)*

*…so having to see a counselor a psychologist would be after my boyfriend and my friends have failed to deal with my problem. (Participant 4)*

The earlier excerpts indicate that students lacked awareness of the likely benefits of mental health services. Instead, the participants suggest that there are better coping strategies besides MHS. Participants minimized the importance of seeking professional counsel because they showed a level of ignorance and awareness of the importance of professional services.

#### Misinformation

The study revealed that participants lacked correct information with regard to mental health services. Because of their embraced distorted information, students are of the view that MHS is not priority intervention measure. This is revealed by the quotes later:

*The thought of going to talk about my life might actually uncover some of the problems in my life that I overcame and that can make me become more stressed. So, whenever I hear mental health issues, I just move away… (Participant 1)*

*…mental disorders are for white people only, it does not apply to us African people, so that’s why I don’t believe in suffering mentally… (Participant 3)*

A general belief by the participants was that mental illness is class related and for particular races in the community. The view was that sharing personal information with a stranger was a weakness rather than a healing process.

### Category 2: Attitudes toward MHS

#### Apathetic

The study’s findings indicate that participants did not appreciate the value and importance of MHS. This position held by the students could be a result of their harbored thoughts that MHS was designed for other people and not themselves. The statements later are illustrative;

*…Personally, I don’t know what it feels like to be stressed, I have never been stressed and so I am strong and I don’t think someone like me will need to seek counselling, there are worse people out there who need counselling not me… (Participant 13)*

*…I don’t want to hear about psychological issues or having to go and see a counsellor, it really makes me sick. (Participant 6)*

It is determined from this study that participants’ personal convictions impacted their evaluation of MHS as not being important.

#### Lack of trust

Trust issues emerged from this study’s findings, wherein it was revealed that participants did not have confidence in the providers of MHS. Students maintained that:

*…I do not trust anyone. If I do not trust my mom and dad why would I trust someone I am not even related to? I have been disappointed by people I trust in my life and trusting someone I do not know is not something I will ever do, I tell my stories to God whenever I face challenges… (Participant 4)*

*…I have been to a group therapy before and a few days after I attended a session, I heard people in the community whispering my name about something I had said in the session and it broke my trust. Therefore, I will never trust anyone in the counselling session including the counsellor… (Participant 12)*

It is evident from the study results that the lack of trust in the service providers leads to negative attitudes toward MHS.

### Category 3: Uptake of mental health services

#### Gender differences

The uptake of MHS has a gender matrix. Apparently, the study showed that as compared with male participants, female participants are ready to take up MHS whenever the need arises. Some of the female participants had this to say:

*I know of the importance of mental health services and l would love to be a psychologist one day, however at the most l cannot afford psychological services and I always have mother (Church Pastor’s wife) to listen to my issues if l have a problem. (Participant 18, female)*

*I am open to seeking psychological help, I have seen the power it has since my best friend attempted suicide and the school counsellor intervened. She is now positive and no longer wants to commit suicide. Since then, I always visit the school counselor when I feel depressed and she is a very nice lady. (Participant 5)*

On their own admission, male participants further supported the finding that female participants have good health seeking behaviors, as evidenced by their resolve to look for MHS. The quotation later illustrates this finding:

*Seeking counseling is for women, men carry bottle their issues lest they lose their dignity. (Participant 1)*

*As males this thing of going to see someone to cry and talk to is taboo it takes away my masculinity (Participant 8)*

*Rather suffer in silence than to seek services from people I do not know as young man we are told by our parents that man don’t cry (Participant 4)*

From the study, it can be seen that female participants have good health-seeking behavior as compared with their male counterparts.

#### Alternative support

The study’s revelation is that participants have other pillars of support apart from the support given by MHS providers. The study participants had this to say:

*Where I come from, we do not tell anyone who is outside of the family anything we are going through both on a personal and family level. Whenever we have challenges in life, we find someone we are close to in the family, we tell them and we get help…. (Participant 2)*

*…Whenever I face a problem, my boyfriend is there to comfort me because I do not trust anyone even my friends…. (Participant 12, female)*

*…My religion does not allow me to seek medical attention or even to go for counselling, if anyone has any challenges it comes out at church and we are given the solution…. (Participant 3)*

Based on the findings mentioned earlier, it is apparent to note that the participants depend on their significant others for support. Hence, the importance of social capital cannot be over-emphasized. It was observed that most students perceived that they had enough social support in their families, friends, and other social circles and, thus, felt that they need not seek opinions from strangers.

## Discussion

The current study revealed that participants lacked correct information about MHS and its related utility. Munson et al. (2009) agrees by stating that some adolescents held that their mental health challenges such as mood disorders were chronic, and as such, they believed that any form of intervention could not control or remedy their illness. Therefore, the information deficit exhibited by secondary students has a corresponding impact on their help-seeking behaviors.

Participants in this study displayed adverse attitudes toward MHS. For example, they exhibited an apathetic attitude toward MHS. In concurrence, Javed et al. (2006) go further to highlight potential reasons for negative attitudes including the style of upbringing and inadequate information about the illness (Dessoki and Hifnawy, 2009).

This study noted a gender disparity with regard to the uptake of MHS by secondary students. The study revealed that, when compared to their male counterparts, the study revealed that female participants are prepared and eager to utilize MHS if need arises. This finding concurs with observations from various scholars who state that despite having mental health needs, male participants are reluctant to utilize MHS (Gonzalez et al., 2005; MacKenzie et al., 2006; Burke et al., 2008). The marked differences in how male and female participants are socialized could be one reason to account for differences in help-seeking behaviors between the two genders, wherein, for example, male participants are socialized to be tough and to “heal” on their own (Munson et al., 2009), and hence their subsequent poor help-seeking behaviors.

## Conclusion

The present study was an investigation of the knowledge, attitudes, and uptake of mental health services by secondary school students in Gweru, Zimbabwe. Participants displayed incorrect information about mental illnesses and subsequent MHS. Various factors adversely affected the attitudes of secondary school students toward MHS. On the uptake of MHS, the study highlights gender differences where female participants display good help-seeking behaviors as compared with their male counterparts.

The study recommends psycho-education sessions aimed at equipping secondary students with evidence-based information on mental health challenges and available services. It is hoped that equipping students with appropriate information will positively impact their attitudes and resultantly lead to the uptake of MHS by both male and female participants as and when the need arises.

## Limitations and future research

This study was located in urban schools limits the generalization of the findings to other contexts. A quantitative study is proposed so as to determine the association between knowledge, attitudes, and uptake of mental health services. The association between the three components is envisaged to further illuminate the subject under the study.

## Data availability statement

The raw data supporting the conclusions of this article will be made available by the authors, without undue reservation.

## Ethics statement

The studies involving human participants were reviewed and approved by Midlands State University Ethics Committee. Written informed consent to participate in this study was provided by the participants’ legal guardian/next of kin.

## Author contributions

SK prepared the first draft of the manuscript. KK reviewed the manuscript. RS collected data. IS analyzed the findings and reviewed the manuscript. TS analyzed the findings and reviewed the manuscript. All authors contributed to the article and approved the submitted version.

## Conflict of interest

The authors declare that the research was conducted in the absence of any commercial or financial relationships that could be construed as a potential conflict of interest.The handling editor TS and reviewer MM declared a shared parental affiliation with the Author SK at the time of the review.

## Publisher’s note

All claims expressed in this article are solely those of the authors and do not necessarily represent those of their affiliated organizations, or those of the publisher, the editors and the reviewers. Any product that may be evaluated in this article, or claim that may be made by its manufacturer, is not guaranteed or endorsed by the publisher.
